# Involvement of Protein Kinase CgSat4 in Potassium Uptake, Cation Tolerance, and Full Virulence in *Colletotrichum gloeosporioides*

**DOI:** 10.3389/fpls.2022.773898

**Published:** 2022-04-07

**Authors:** Yu-Ting Pan, Lianwei Li, Ji-Yun Yang, Bing Li, Yun-Zhao Zhang, Ping Wang, Lin Huang

**Affiliations:** ^1^Co-Innovation Center for Sustainable Forestry in Southern China, Nanjing Forestry University, Nanjing, China; ^2^The Key Laboratory of Biotechnology for Medicinal Plants of Jiangsu Province, School of Life Science, Jiangsu Normal University, Xuzhou, China; ^3^School of Agricultural Sciences, Zhengzhou University, Zhengzhou, China

**Keywords:** anthracnose, pathogenicity, phosphorylation, potassium accumulation, protein kinase

## Abstract

The ascomycete *Colletotrichum gloeosporioides* is a causal agent of anthracnose on crops and trees and causes enormous economic losses in the world. Protein kinases have been implicated in the regulation of growth and development, and responses to extracellular stimuli. However, the mechanism of the protein kinases regulating phytopathogenic fungal-specific processes is largely unclear. In the study, a serine/threonine CgSat4 was identified in *C. gloeosporioides*. The CgSat4 was localized in the cytoplasm. Targeted gene deletion showed that CgSat4 was essential for vegetative growth, sporulation, and full virulence. CgSat4 is involved in K^+^ uptake by regulating the localization and expression of the potassium transporter CgTrk1. CgSat4 is required for the cation stress resistance by altering the phosphorylation of CgHog1. Our study provides insights into potassium acquisition and the pathogenesis of *C. gloeosporioides*.

## Introduction

Protein phosphorylation catalyzed by protein kinases is essential for the regulation of growth, development, and responses to extracellular stimuli in eukaryotic cells (Wang et al., 2011; Chen et al., 2017). Based on the catalytic domains, protein kinases have been divided into several families, including the STE (homologs of yeast Sterile 7, Sterile 11, Sterile 20 kinases), CK1 (casein kinase 1), CAMK (Ca^2+^/calmodulin-dependent protein kinase), CMGC (cyclin-dependent, mitogen-activated, glycogen synthase, and cyclin-dependent protein kinase-like kinases), AGC (protein kinase A, G, and C families), HisK (histidine kinase), RGC (receptor guanylate cyclase kinases), TK (tyrosine kinases), TKL (tyrosine like kinases), atypical families, and others (Miranda-Saavedra and Barton, 2007; De Souza et al., 2013). Several transcription factors, enzymes, membrane proteins including transporters and ion channels, and other kinases have been identified as the substrates of protein kinases and have been found to be regulated by phosphorylation (Smith et al., 2010; Turrà et al., 2014; Martin et al., 2015). In phytopathogenic fungi, expansion of kinases was considered to be beneficial for the pathogen to adapt to stresses encountered both external and internal of its host (Deiulio et al., 2018).

Potassium is essential for maintaining the cell shape, membrane potential, intracellular pH, and enzyme activity (Pérez-Valle et al., 2007; Kahm et al., 2012). Potassium acquisition against a concentration gradient into the cell is derived by conserved families of proteins TRK (derived from the Transporter of K^+^), HAK (derived from High-Affinity K), ACU ATPases (derived from Alkali Cation Uptake transporters), and PAT (derived from P-type ATPase) in fungi (Benito et al., 2004, 2011; Corratgé et al., 2007; Haro and Benito, 2019). In the yeast *Saccharomyces cerevisiae*, the protein kinases Hal4, Hal5, and Sky1 have been determined as regulators to involve in potassium uptake (Rodríguez-Navarro, 2000; Wang et al., 2005; Pérez-Valle et al., 2007). The serine/threonine kinase Sat4 (Hal4) positively regulates potassium influx by stabilizing potassium transporters Trk1 and Trk2 (Mulet et al., 1999; Hirasaki et al., 2011). The serine/threonine kinase Sat4 is one of the CAMK kinases. The deletion of *SAT4* led to significantly reduced K^+^ in the yeast cells and increased sensitivity to NaCl, LiCl, and CaCl_2_ (Mulet et al., 1999). The orthologs of Sat4 have been identified in the filamentous fungi (Wang et al., 2011; De Souza et al., 2013; Yang et al., 2018). *Fusarium graminearum* FgSat4 was required for vegetative growth, sporulation, conidial morphology, and pathogenicity (Wang et al., 2011). In *Colletotrichum higginsiaum*, ChSat4 was determined to be involved in cell wall integrity, hyperoxide stress response, and pathogenicity (Yang et al., 2018). These studies have reported the biological phenotype of Δ*sat4* in fungi, but the specific molecular mechanism of Sat4 regulating the pathogenicity as a protein kinase in plant pathogenic fungi remains to be studied.

The ascomycete *Colletotrichum gloeosporioides* employs a hemibiotrophic strategy to infect the plant hosts and causes enormous economic losses to crop production and forest industry worldwide (Dean et al., 2012; Wang et al., 2020). Anthracnose caused by *C. gloeosporioides* is one of the most serious diseases on *Cunninghamia lanceolata* (Huang et al., 2019). The *C. gloeosporioides* genome has been sequenced and 16,287 protein-coding genes were identified (Huang et al., 2019). Several genes encoding laccases, peroxidases, plant cell wall-degrading enzymes, Cytochrome P450, and secretory protein have been predicted, which were considered potential pathogenicity contributors in other phytopathogenic fungi (Dean et al., 2005; Yin et al., 2015). In addition, a large number of protein kinases have been predicted. Several protein kinases, such as CgRhoB, CgSte50, CgSte11, CgSte7, CgMk1, CgMck1, have been proved to play important roles in the growth, development, reproduction, and pathogenicity in *C. gloeosporioides* (Xu et al., 2016; He et al., 2017; Fang et al., 2018; Wang et al., 2021). However, despite these advances, the majority of protein kinases await functionally characterization.

In this study, a yeast serine/threonine kinase Sat4 homolog CgSat4 was identified and characterized in *C. gloeosporioides*. Our data showed that CgSat4 is involved in K^+^ uptaking by regulating the accurate localization and expression of the potassium transporter CgTrk1. CgSat4 is required for the osmotic resistance by altering the phosphorylation level of high osmolarity glycerol response kinase CgHog1 that plays important role in the normal response to hyperosmotic stress and cation stress. Deletion of the *CgSAT4* also resulted in defects of vegetative growth, sporulation, and pathogenicity in *C. gloeosporioide*.

## Materials and Methods

### Fungal Strain and Culture Conditions

*Colletotrichum gloeosporioides* strain SMCG1#C (Huang et al., 2019) was used for the wild type (WT). The WT, gene deletion mutants, and the complemented strains were maintained on the potato dextrose agar (PDA) plates at 25°C. Complete medium (CM) was employed to culture fungal mycelia for DNA extraction and protoplast preparation as aforedescribed (Wang et al., 2020).

### Mutagenesis of *CgSAT4* and Complementation of the Mutant

The *CgSAT4* was identified in the *C. gloeosporioides* genome database^1^ using BLASTP with amino acid sequences of Sat4/Hal4 (NM_001178721.1) from *S. cerevisiae*. Protein sequences were aligned with ClustalX 2.1, and a phylogenetic tree of the CgSat4 and its orthologs from different fungi was generated using MEGA 6.0 (Tamura et al., 2013).

The knockout deletion mutant of *CgSAT4* was obtained using the method described by Wang et al. (2020). Firstly, the upstream and downstream flanking regions (*ca.* 1.2 kb) flanking sequences of *CgSAT4* were amplified using the primer sets of 1F/2R and 3F/4R (Supplementary Table S1), respectively. The PCR products were ligated to either side of the hygromycin B phosphotransferase (*HPH*) cassette to generate the gene replacement fragment using overlap PCR with the primer set OuterF/OuterR (Supplementary Table S1). Secondly, the gene replacement fragments were purified and transformed into the protoplasts of the WT according to the aforedescribed procedure (Yang et al., 2018). Thirdly, the candidate transformants were screened on the TB3 plate and confirmed by Southern blotting as described previously (Wang et al., 2020), with the probe generated with the primer sets *CgSAT4*_SN_F/*CgSAT4*_SN_R and F1111/F1112 (Supplementary Table S1), respectively.

For complementation, a 3.0-kb fragment containing the *CgSAT4* ORF region and its native promoter (~1,500 bp) was amplified with the primers *CgSAT4* -GFP-1F and *CgSAT4*-GFP-2R (Supplementary Table S1). The PCR products were inserted into the vector pYF11 that was linearized with the restriction endonuclease *Xho* I using the yeast gap repair approach (Zhou et al., 2011). The resulting fusion construct *CgSAT4*-GFP was verified by sequencing and transformed into the *CgSAT4* gene deletion mutant. The positive complemented strains were screened by GFP signals and Western blotting described by Bruno et al. (2004).

### Localization Pattern Analyses of CgSat4

To observe the localization of CgSat4, mycelial plugs of the complemented strain Δ*Cgsat4/SAT4* expressing fusion protein CgSat4-GFP were cultured in liquid CM medium at 25°C for 24 h. Then, fresh mycelia of the corresponding strains expressing fusion protein CgTrk1-GFP were prepared for fluorescence microscopy observation as described above. Photographs were taken under a confocal laser scanning microscope (Zeiss, Oberkochen, Germany). To evaluate the effect of deletion of *CgSAT4* on the localization of CgTrk1, the construct of *CgTRK1*-GFP was introduced into the WT and the Δ*Cgsat4* mutant, respectively, as described above. The localization of CgTrk1 in these strains were observed under a confocal laser scanning microscope. The experiment was performed twice.

### Assays of Vegetative Growth and Fruiting Bodies Development

For vegetative growth assays, the mycelial blocks (6 mm in diameter.) of the WT, the Δ*Cgsat4* mutant, and the complemented strain were inoculated onto PDA plates, respectively. The plates were kept in an incubator at 25°C. Colony growth kinetics was measured at 5 days post-inoculation. Fruiting bodies were induced on V8 juice agar (V8) plates for 10 days after inoculation at 25°C according to the method of Fang et al. (2018). The experiment was carried out three times, and each treatment had three replicates.

### Stress Resistance Assays and Determination of Potassium in Fungal Mycelia

To test the role of CgSat4 on stress resistance, the WT, the Δ*Cgsat4* mutant, and the complemented strains were inoculated on CM plates supplemented with NaCl (0.7 M), KCl (0.7 M), or LiCl (0.3 M). These plates were kept at 25°C for 4 days. The experiment was carried out three times, and each treatment had three replicates.

To evaluate the effect of CgSat4 on potassium uptake, the WT and Δ*Cgsat4* mutant were cultured in liquid CM medium containing 7 mM potassium for 2 days as described previously (Yang et al., 2018). The fungal mycelia were harvested and dried in a freeze-dryer. Then, the dried mycelia were digested with H_2_SO_4_, and mycelial potassium was examined using a flame spectrophotometer (Yang et al., 2018). The experiment was conducted three times, and each treatment had three replicates.

### Sporulation, Appressorium Formation, and Invasive Hypha Development

For sporulation, the mycelial blocks (6 mm in dia.) of the WT, the Δ*Cgsat4* mutant, and the complemented strains were inoculated in the carboxymethyl cellulose (CMC) medium to induce sporulation, and the conidia were collected and counted as described by Fang et al. (2018). The experiment was carried out three times, and each treatment had three replicates.

To induce conidial germination and appressorium formation, the conidial suspensions of the WT, the Δ*Cgsat4* mutant, and the complemented strains were adjusted to 10^5^/ml, respectively. Ten microliters of conidial suspension of each strain was placed on the glass coverslip (Fisher Scientific, St. Louis, MO, United States) and kept at 25°C. The conidial germination rate of each strain was calculated at 2, 4, and 8 h postinoculation, respectively. The percentage of appressorium formation of each strain was tested at 4, 8, and 12 h postinoculation, respectively. Appressorium turgor pressure was analyzed by the incipient cytorrhysis assay using 1–4 M of glycerol solution, as described by Wang et al. (2020). The experiment was conducted three times with at least 100 structures per replicate.

Onion penetration assays were conducted as described by Wang et al. (2020). Ten microliters of conidial suspension of the WT, the Δ*Cgsat4* mutant, and the complemented strains was inoculated on the adaxial surface of onion epidermal strips to induce invasive hyphae (IH), respectively. At 24 h post-inoculation, IH was observed under a Zeiss Axio Imager A2M microscope (Carl Zeiss, Jena, Germany). IH was divided into four types (type I, no hyphae penetration; type II, IH with one branch; type III, IH with at least two branches, but having limited expansion; type IV, IH with numerous branching and extensive hyphal growth). The experiment was performed three times, and at least 30 invasive structures were observed in each treatment.

### Pathogenicity Tests

Pathogenicity of the WT, the Δ*Cgsat4* mutant, and the complemented strain was tested as described by Wang et al. (2020). Conidial suspensions of the WT, the Δ*Cgsat4* mutant, and the complemented strains were adjusted to 1 × 10^5^ spores/ml, respectively. Five microliters of conidial suspension of each strain was inoculated on healthy leaves of *C. lanceolata*, *Populus × euramericana* cv. *“Nanlin895”* and *Liriodendron chinense × tulipifera*, respectively. The inoculated leaves were kept in a moist chamber at 25°C, and lesion size was measured at 5 days post-inoculation. DNA was isolated from the *L. chinense × tulipifera* leaves inoculated by the WT and the Δ*Cgsat4* mutant, and the fungal biomass *in planta* was examined using qPCR as described by Yang et al. (2018). The experiment was performed three times, and each treatment had three replicates.

### Protein Expression, Protein Extraction, and Western Blot

Mycelial plugs of the WT and the Δ*Cgsat4* mutant were inoculated into 100 ml of CM medium, and shaken at 150 rpm for 2 days at 25°C, respectively. The mycelia of each strain were collected using a layer of Miracloth. Total protein was extracted from the mycelia following the method described by Bruno et al. (2004). Twenty microliters of total proteins were isolated on the SDS-PAGE gel and transferred to a polyvinylidene fluoride (PVDF) membrane using a Bio-Rad imprinting device (Bio-Rad Laboratories, Inc., CA, United States). A primary anti-GFP antibody (GFP labeled mouse monoclonal antibody, Shanghai Antibody Market, China) and a secondary antibody (goat anti-mouse IgG horseradish peroxidase, Shanghai Antibody Market, China) were used to detect GFP. Signal strength corresponding to phosphorylated Hog1 was detected by binding of anti-phosphorylated p38 MAPK (Thr180/Tyr182, rabbit monoclonal antibody) (Cell Signaling Technology, Boston, MA, United States). P38 MAPK antibody (ABMART) was used as the control. The experiment was carried out twice.

To evaluate the effect of the CgSat4 on the potassium transporter CgTrk1 localization and expression, the *CgTRK1* coding region, and its native promoter sequence (~1,500 bp) were amplified with the primers *CgTRK1*-GFP-1F and *CgTRK1*-GFP-2R. The PCR products were purified and inserted into the vector pYF11 as aforedescribed (Zhou et al., 2011). The fusion construct *CgTRK1*-GFP was transformed into the WT and the Δ*Cgsat4* mutant, respectively. The positive candidate strains expressing fusion protein CgTrk1-GFP were screened by GFP signals observation. The expression levels of CgTrk1-GFP in the WT and the Δ*Cgsat4* mutant were detected using Western blotting with anti-GFP antibody as aforedescribed. The experiment was carried out twice.

### Phos-tag Analysis

The *CgTRK1-*GFP fusion construct was transferred, respectively, into the wild-type strain and ∆*Cgsat4* mutant. The positive transformants were cultured in liquid CM for 48 h. For protein isolation, about 150–200 mg of mycelia were ground into powder in liquid nitrogen and resuspended in 1 ml of extraction buffer [10 mM Tris–HCl (pH 7.5), 150 mM NaCl, 0.5 mM EDTA, 0.5% NP40, 1 mM PMSF, 10 μl of protease inhibitor cocktail (Sigma, United States), and 10 μl of phosphatase inhibitor cocktail 3 (Sigma, United States)]. For the preparation of the phosphatase-treated Cell lysates, the phosphatase inhibitor cocktail was omitted for 2.5 U/ml alkaline phosphatase (final concentration; P6774; Sigma) and the sample was incubated for 1 h with the addition of 1 mM MgCl2 (37°C). The samples were then resolved on a 8% SDS-polyacrylamide gel prepared with 50 μM acrylamide-pendant Phos-tag ligand (Wako, Japan) and 100 μM MnCl_2_, according to the instruction provided by the Phos-tag.

### Quantitative Real-Time PCR, and Statistical Analyses

To evaluate the effect of deletion of *CgSAT4* on the transcription level of CgTrk1, the mycelia of the WT and the Δ*Cgsat4* mutant were, respectively, cultured in liquid CM for 3 days at 25°C. The mycelia were collected and total RNA was extracted using the TRIzol LS reagent (Invitrogen, Carlsbad, CA, United States). The first-strand cDNA was synthesized and used as the templates of quantitative RT-PCR as described by Yang et al. (2018). The experiment was conducted three times, and each treatment had three replicates.

Data are presented as mean ± SD. Statistical analyses were carried out with the data processing system (DPS) version 9.50 using a one-way ANOVA (*p* < 0.01).

## Results

### Identification of CgSat4 and Gene Deletion Mutant of *CgSAT4*

An orthologue of Sat4 was identified by a BLAST_P search using the Sat4/Hal5 (NM_001178721.1) of *S. cerevisiae* as the reference to the genome database of *C. gloeosporioides*.^2^ The orthologue shared a 65% amino acid sequence identity with *S. cerevisiae* Sat4, which was named CgSat4. Phylogenetic analysis showed that Sat4 anthologies in filamentous fungi have significantly diverged from that of *S. cerevisiae*. The CgSat4 was most similar to its orthologues from phytopathogenic fungi of *Colletotrichum* species (Supplementary Figure S1A). CgSat4 contained a low complexity region and a Pfam Pkinase motif at the C-terminus (Supplementary Figure S1B).

A gene deletion mutant Δ*Cgsat4* was obtained by replacing the open reading frame with the hygromycin phosphotransferase resistance (*HPH*) gene (Supplementary Figure S1C). Southern blotting analysis confirmed that the *CgSAT4* was encoded by a single-copy gene in the WT, which was replaced by the *HPH* gene in the Δ*Cgsat4* mutant (Supplementary Figure S1D). A complemented strain Δ*Cgsat4/CgSAT4* was generated by reintroducing the *CgSAT4* encoding region with its native promoter into the Δ*Cgsat4* mutant.

To investigate the localization pattern of CgSat4 in *C. gloeosporioides*, the CgSat4-GFP fusion construct was introduced into the Δ*Cgsat4* mutant to generate the complemented strain Δ*Cgsat4/CgSAT4*. The complemented strain recovered the defects of the *CgSAT4* deletion mutant as described above, which indicated the CgSat4 has been properly expressed. Thus, the GFP signals of the complemented strain were employed to determine the localization of CgSat4. The fluorescence microscopy showed that strong GFP signals were distributed in the cytoplasm of the conidia and hyphae (Supplementary Figure S1E). Furthermore, an 80-kDa of the predicted CgSat4-GFP fusion protein was detected using an anti-GFP antibody (Supplementary Figure S1F). The data indicated that CgSat4 was expressed in the cytoplasm of *C. gloeosporioides*.

### CgSat4 Required for Vegetative Growth, Fruiting Body Development, and Sporulation

**Figure 1:**
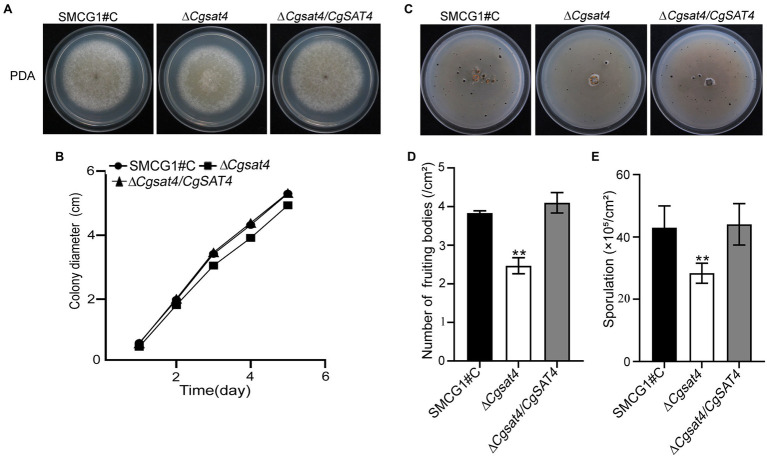
Involvement of CgSat4 in vegetative growth, fruiting body development and sporulation. **(A)** Colonies of the WT (SMCG1#C), ∆*Cgsat4* mutant and the complemented strain ∆*Cgsat4/CgSAT4* were cultured on potato dextrose agar (PDA) at 25°C for 5 days. *n* = 3. **(B)** Colony growth kinetics in **(A)**. *n* = 3. **(C)** Fruiting bodies of the WT, ∆*Cgsat4* mutant and the complemented strain formed on the V8 medium for 10 days with 16 h light/8 h dark cycle. *n* = 3. **(D)** Quantitative statistics of fruiting bodies in **(C)**. *n* = 3. **(E)** Sporulation of the WT, ∆*Cgsat4* mutant and the complemented strain. *n* = 3. Error bars represent the SD, and asterisks indicate significant difference at *p* < 0.01.

To assess the role of CgSat4, the WT, the Δ*Cgsat4* mutant, and the complemented strain Δ*Cgsat4/CgSAT4* were, respectively, inoculated on the PDA. The growth kinetics was observed. On the 4th and 5th day, the colony diameter of the Δ*Cgsat4* mutant significantly reduced compared to the WT and complemented strains (Figures 1A,B).

Fruiting body quantification showed that fruiting bodies produced by the Δ*Cgsat4* mutant were significantly less than those of the WT and complemented strains (Figures 1C,D). There are no morphological differences in the conidia among the WT, Δ*Cgsat4* mutant, and complemented strain Δ*Cgsat4/CgSAT4*. However, conidial enumeration showed that conidia produced by the Δ*Cgsat4* mutant were considerably less than those of the WT and complemented strain (Figure 1E). These results indicated that CgSat4 is required for vegetative growth, fruiting body development, and sporulation in *C. gloeosporioides*.

### CgSat4 Required for Potassium Uptake

**Figure 2:**
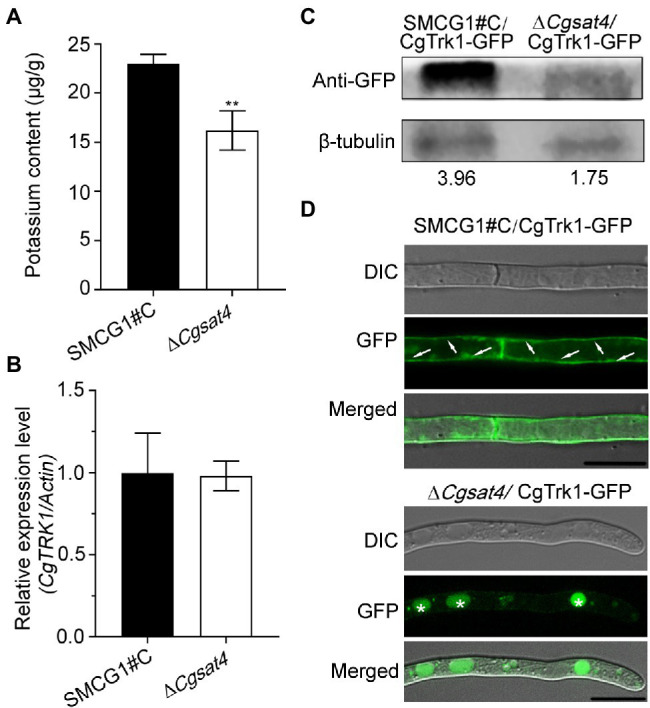
CgSat4 required for potassium uptake and the localization of potassium transporter CgTrk1. **(A)** Mycelia K^+^ concentration of the WT and ∆*Cgsat4* mutant cultured in CM medium. *n* = 3. **(B)** Transcription level analysis of *CgTRK1* in the WT and ∆*Cgsat4* mutant using qRT-PCR. **(C)** Western blotting analysis of CgTrk1 in the WT and ∆*Cgsat4* mutant using an anti-GFP antibody. **(D)** Localization pattern of the fusion protein CgTrk1-GFP in the WT and ∆*Cgsat4* mutant. The arrows and asterisks represent the plasma membrane and vacuole, respectively. Error bars represent the SD, and asterisks indicate significant difference at *p* < 0.01.

In the yeast *S. cerevisiae*, the Sat4 is involved in potassium influx which is mediated by the Trk1-Trk2 transport system (Mulet et al., 1999; Hirasaki et al., 2011). To evaluate whether the orthologue *C. gloeosporioides* CgSat4 is required for potassium uptake, the mycelial potassium content of the WT and the Δ*Cgsat4* mutant was examined. Data showed that the potassium was significantly reduced in the Δ*Cgsat4* mutant than in the WT (Figure 2A).

The transcription level of CgTrk1 was detected in the WT and Δ*Cgsat4* mutant by qRT-PCR. The result showed that there was no significant transcription difference of *CgTRK1* between the WT and Δ*Cgsat4* mutant (Figure 2B). However, western blotting analysis showed the expression of CgTrk1 in WT was more than twice as much as that in the Δ*Cgsat4* mutant (Figure 2C).

The localization pattern analysis also showed that the potassium transporter CgTrk1 was distributed in the plasma membrane in the WT. However, the CgTrk1 was mainly distributed in the vacuoles in the Δ*Cgsat4* mutant (Figure 2D). We therefore examined the interaction between CgSat4 and CgTrk1 by co-immunoprecipitation (co-IP) and yeast two-hybrid (Y2H) assays. However, the results show that CgSat4 does not interact directly with CgTrk1 (Supplementary Figures S2A,B). We also examined whether CgSat4 regulates the level of CgTrk1 phosphorylation through Phos-tag SDS-PAGE gel electrophoresis. The shifts in the mobility of CgTrk1-GFP from the wild-type strain and ∆*Cgsat4* mutant cells had no obvious difference (Supplementary Figure S2C), suggesting that CgSat4 does not regulate the level of CgTrk1 phosphorylation. Combining the aforementioned results, we concluded that CgSat4 may be involved in potassium uptake by regulating the localization of the high-affinity potassium transporter CgTrk1.

### CgSat4 Required for Extracellular Ion Stress Resistance

**Figure 3:**
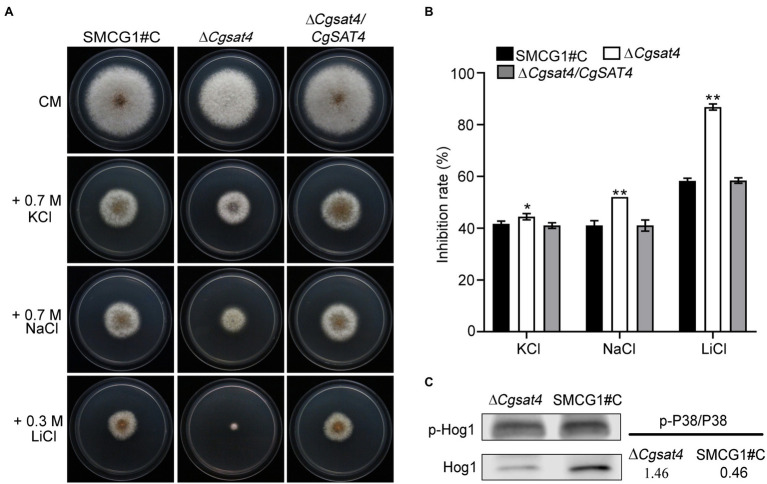
CgSat4 required for extracellular cation stress resistance. **(A)** Colonies of the WT, ∆*Cgsat4* mutant, and the complemented strain ∆*Cgsat4/CgSAT4* cultured on CM medium supplemented with different cations for 4 days. *n* = 3. **(B)** Colony diameter inhibition rate in **(A)**. Error bars represent the SD. * and ** indicate significant difference at *p* < 0.05 and 0.01, respectively. *n* = 3. **(C)** Phosphorylation level of CgHog1 in the WT and ∆*Cgsat4* mutant was detected using an antiphospho-p38 antibody.

In eukaryotic cells, the Hog1 mitogen-activated protein kinase (MAPK) pathway is essential to osmotic stress response (Brewster et al., 1993; Román et al., 2020). As an intracellular osmotic stress regulator, potassium participates in various physiological processes (Pérez-Valle et al., 2007; Kahm et al., 2012). Since the deletion of *CgSAT4* resulted in the decrease of mycelial potassium, we hypothesized that decreased potassium concentration may alter the intracellular osmotic pressure and resistance against osmotic stress. To verify this hypothesis, the WT, Δ*Cgsat4* mutant, complemented strain Δ*Cgsat4/CgSAT4* were, respectively, inoculated onto the CM plates subjected to osmatic stressors KCl, NaCl, and LiCl, respectively. Compared with the WT and complemented strains, the Δ*Cgsat4* mutant was more sensitive to the osmatic stressors and displayed a higher growth inhibition rate (Figures 3A,B). The effect of *CgSAT4* deletion on the phosphorylation of CgHog1 was further evaluated. The result showed that deletion of *CgSAT4* significantly increased the phosphorylation level of CgHog1 compared to the WT (Figure 3C). However, an interaction between CgSat4 and CgHog1 cannot be reproduced (Supplementary Figure S2D).

### CgSat4 Is Required for Functional Appressorium and Invasive Hyphal Development

**Figure 4:**
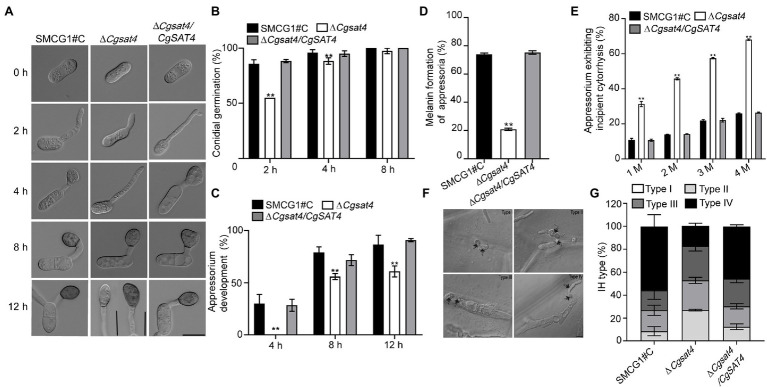
CgSat4 is required for functional appressorium and invasive hyphal development. **(A)** Conidia and appressoria generated by the WT, ∆*Cgsat4* mutant and the complemented strain on hydrophobic slides at 0, 2, 4, 8, and 12 h and photographed. *n* = 100. Bar = 10 μm. **(B)** Conidial germination rate of the WT, ∆*Cgsat4* mutant and the complemented strain at 2, 4, and 8 h, respectively. *n* = 100. **(C)** Appressorium formation rate of the WT, ∆*Cgsat4* mutant and the complemented strain at 4, 8, and 12 h, respectively. Error bars represent the SD. Asterisks indicates significant difference at *p* < 0.01. *n* = 100. **(D)** Melanized appressoria rate of the WT, ∆*Cgsat4* mutant, and complemented strain. *n* = 100. **(E)** Collapsed appressoria rate of the WT, ∆*Cgsat4* mutant, and complemented strain. *n* = 100. Error bars represent the SD. Asterisks indicates significant difference at *p* < 0.01. **(F)** Morphology of different types of invasive hypha developed on onion epidermal cells for 24 h. *n* = 100. **(G)** Proportion of different type of invasive hypha (type I, no hyphae penetration; type II, IH with one branch; type III, IH with at least two branches, but having limited expansion; type IV, IH with numerous branching and extensive hyphal growth) in the WT, ∆*Cgsat4* mutant and complemented strain. Error bars represent the SD. *n* = 100. Bar = 10 μm.

We found that deletion of *CgSAT4* did not affect the conidial morphology. Compared with the WT and complemented strains, the conidial germination rate of the Δ*Cgsat4* mutant significantly decreased at 2 and 4 h. However, there was no significant difference in the conidial germination among the WT, Δ*Cgsat4* mutant, complemented strains at 8 h (Figures 4A,B). These data indicate that deletion of the *CgSAT4* significantly delayed conidial germination in the early stage.

The data showed that, compared with the WT and complemented strains, appressorium formation rate of the Δ*Cgsat4* mutant was significantly decreased (Figure 4C). In the Δ*Cgsat4* mutant, 80% of appressoria failed to melanize, while the WT and the complemented strains formed normal melanized appressoria (Figure 4D). At different concentrations of glycerol, the appressorium collapse rate of the Δ*Cgsat4* mutant was significantly higher than those of the WT and the complemented strains (Figure 4E). These results indicated that deletion of the *CgSAT4* caused the defects in appressorium formation and appressorial turgor pressure.

The onion epidermis penetration assays also showed that the percentages of Types I, II, III, and IV infectious hyphal growth of the Δ*Cgsat4* mutant were, respectively, 27%, 25.9%, 29.9%, and 21%, compared with 8.6%, 18.2%, 17.5%, and 55.6% in the WT (Figures 4F,G).

### CgSat4 Required for Full Virulence

**Figure 5:**
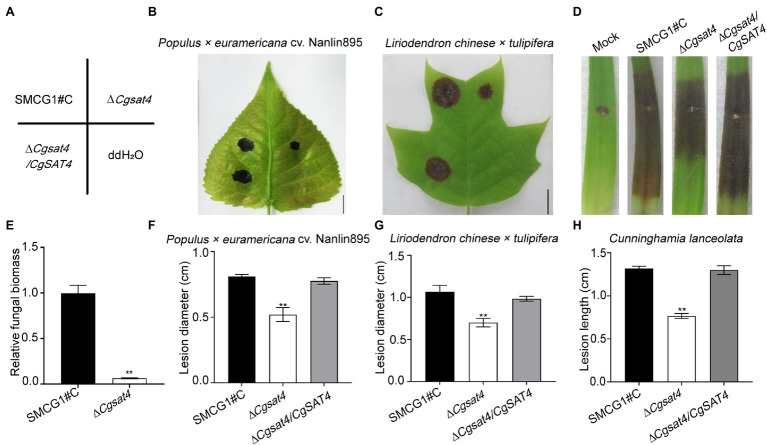
CgSat4 required for full virulence in *C. gloeosporioides*. **(A)** Diagram of inoculation sites of the WT, ∆*Cgsat4* mutant and complemented strain on the unwounded leaves of *Populus × euramericana* cv. Nanlin895 and *Lirioden dronchinensis × tulipifera*. *n* = 3. **(B,C)** Pathogenicity assays on leaves of *Populus × euramericana* cv. Nanlin895 and *L. dronchinensis × tulipifera* for 5 days indicated in **(A)**, respectively. *n* = 3. **(D)** Pathogenicity assays on wounded leaves of *C. lanceolata* for 5 days. *n* = 3. **(E)** Relative fungal biomass of the WT and ∆*Cgsat4* mutant monitored from infected tissues of *L. dronchinensis × tulipifera* at 3 dpi, respectively. *n* = 3. **(F–H)** Lesion diameter of lesions on the inoculated leaves showed in **(B–D)**, respectively. *n* = 3. Error bars represent the SD. Asterisks indicates significant difference at *p* < 0.01.

To explore the role of CgSat4 in host infection, conidial suspensions of the WT, Δ*Cgsat4* mutant, and the complemented strains were, respectively, inoculated on the healthy leaves of *Populus × euramericana* cv. Nanlin895. Five days post-inoculation (dpi), the Δ*Cgsat4* mutant produced smaller lesions than those of the WT and complemented strains (Figures 5A,B,F). Similarly, compared with the WT and complemented strains, the Δ*Cgsat4* mutant showed a decreased virulence on *Liriopendron chinense × tulipifera*, and *Cunninghamia lanceolata* (Figures 5A,C,D,G,H). Fungal biomass assays using the qPCR method also showed that the biomass of the Δ*Cgsat4* mutant *in planta* was significantly reduced than that of the WT (Figure 5E). These data indicated that CgSat4 played a key role in infectious growth and full virulence in *C. gloeosporioides*.

## Discussion

*Colletotrichum gloeosporioides* employs a hemibiotrophic lifestyle (Dean et al., 2012; Yan and Talbot, 2016). The infection of *C. gloeosporioides* is mediated by the appressoria, which drive the penetration peg through leaf cuticles and cell walls (Fang et al., 2018; Wang et al., 2020). In this study, a serine/threonine protein kinase CgSat4 was found to be required for vegetative growth, functional appressorium formation, invasive hyphal development, and full virulence. Further study showed that deletion of *CgSAT4* resulted in defects of potassium uptake and higher sensitivity to salt ions than wild type. In the phytopathogenic fungus *F. graminearum*, the Δ*Fgsat4* mutant also displayed an increased sensitivity to salt ions (Wang et al., 2011). Deletion of *ChSAT4* led to a similar phenotype defect in *C. higginsianum* (Yang et al., 2018). In this study, we found that CgSat4 regulated potassium accumulation, and played an essential role against cation toxicity of high concentration of Na^+^, K^+^, and Li^+^. Since potassium is important for cell growth (Hušeková et al., 2016), the inhibited vegetative and invasive growth of *C. gloeosporioides* in the Δ*Cgsat4* mutant are due to the reduction of potassium accumulation. The abnormal potassium transport caused imbalance of the mutant’s ability to regulate ion stress and osmotic stress. As a hemibiotrophic pathogenic fungus, *C. gloeosporioides* will also be stressed by ions and osmotic pressure from host cells and environment.

Potassium (K^+^) is the most abundant intracellular cation in living cells (Pérez-Valle et al., 2007; Kahm et al., 2012). In fungi, potassium uptake against electrical and concentration gradient is derived by conserved families of proteins Trk, Hak, Acu ATPases, and Pat (Corratgé et al., 2007; Benito et al., 2011; Haro and Benito, 2019). In *S. cerevisiae*, the kinase Sat4/Hal4 and its homolog Hal5 have been determined to involve in ion homeostasis. Overexpression of *SAT4* increased tolerance to sodium and lithium. The mutant lacking *SAT4* displays a higher sensitivity to toxic cations than the wild type. The Trk1 functions as a potassium transporter and facilitates cells to survive in a low potassium environment (Gaber et al., 1988). The potential regulating mechanism study shows that the Trk1-Trk2 potassium transporter increase the influx of potassium, and decrease the membrane potential, which results in reduced the uptake of toxic cations and improved salt tolerance (Mulet et al., 1999). Trk1 has been determined to be localized in the plasma membrane of *S. cerevisiae*, which ensures its normal physiological function (Kale et al., 2019). Our results showed that the localization and protein levels of CgTrk1 are regulated by the CgSat4. The localization of CgTrk1 was changed from the plasma membrane to the vacuole in the mutant Δ*Cgsat4*. The abnormal localization and reduced protein levels of CgTrk1 may suppress the potassium uptake, and result in a significant decrease of potassium accumulation and resistance to ion stress in *C. gloeosporioides*. The difference of CgTrk1 localization in ∆*Cgsat4* mutant may be due to the increased endocytosis of lipid membrane proteins after deletion of *CgSAT4*. In *S. cerevisiae*, the absence of *SAT4* leads to increased internalization of many lipid membrane proteins and receptors of some signaling pathways (Tumolo et al., 2020). In addition, in phytopathogenic fungi, endocytosis also has been charactered as a determiner of virulence (Fuchs et al., 2006). Lipid membrane proteins that complete signal transmission often enter the vacuole and are degraded. The CgTrk1 was mainly distributed in the vacuoles in the Δ*Cgsat4* mutant. The lower protein levels for CgTrk1 in Δ*Cgsat4* mutant may be due to more degradation of CgTrk1 in Δ*Cgsat4* mutant.

The high osmolarity glycerol (HOG) pathway required for osmoregulation depends on the mitogen-activated protein kinase (MAPK) Hog1 cascade, which is highly conserved from single-cell yeast to filamentous fungi (Hohmann, 2009; de Assis et al., 2020; Román et al., 2020). Hog1 induces cellular response to high osmolarity stress in *S. cerevisiae* (Brewster et al., 1993; Román et al., 2020). In this study, deletion of *CgSAT4* significantly altered the phosphorylation level of CgHog1 and sensitivity to osmotic stress. In the fission yeast *Schizosaccharomyces pombe*, the Hog1 ortholog, Spc1, is responsive to multiple environmental stresses (Shiozaki and Russell, 1995; Kato et al., 1996). Proper phosphorylation and activation of Spc1 is required for activation of transcription factor Atf1 that induce the expression of genes involved in resistance against various stress conditions (Shiozaki and Russell, 1995; Gaits et al., 1998). Spc1 regulates the expression of a plasma membrane Na^+^/H^+^ antiporter *SOD2* to export Na^+^ and Li^+^ (Jia et al., 1992), which is required for cellular survival of cation toxicity. Deletion of *SAT4* also shows hypersensitivity to cation stress in *S. pombe* (Wang et al., 2005). So the hypersensitivity of ∆*Cgsat4* mutant to osmotic pressure may be due to the inappropriate phosphorylation of CgHog1. The increased phosphorylation level of CgHog1 in ∆*Cgsat4* mutant may be caused by the increased internalization of membrane receptor protein upstream of Hog1 MAPK signaling pathway. In *S. cerevisiae*, the absence of *SAT4* leads to increased internalization of many lipid membrane proteins and receptors of some signaling pathways, such as cell wall stress response protein Wsc1 that is a receptor of MAPK pathway (Tumolo et al., 2020). Combining these data, we propose that CgSat4 is necessary for cation stress resistance *via* balancing the phosphorylation levels of the CgHog1, which is a key component of the high-osmolarity pathway. In fact, in *Magnaporthe oryzae*, another plant hemibiotrophic fungi similar to *C. gloeosporioides*, several conserved signaling pathways are essential for appressorium formation and pathogenicity, including cAMP signaling, Hog1, Pmk1, and Mps1 MAP kinase pathways (Li et al., 2012). In *C. gloeosporioides*, the homologue MAPK pathway kinases CgPka, CgMek1/CgMkk1, CgSlt2/CgMps1, and CgMck1 also have been determined to be involved in the appressorium formation and pathogenicity (Kim et al., 2000; Priyatno et al., 2012; Yong et al., 2013). These results indicated that the invasive growth and pathogenicity of *C. gloeosporioides* were regulated by various complex gene networks.

In summary, we have identified and discussed the potential roles of S/T protein kinase CgSat4 in growth, developmental, environmental stress responses, and pathogenicity. The findings will help illuminate the underlying mechanisms of potassium uptake and its functions in plant infection of *C. gloeosporioides*.

## Conclusion

CgSat4 is required for K^+^ uptake by regulating the localization of the potassium transporter CgTrk1 and cation stress resistance by altering the phosphorylation of CgHog1, and full virulence.

## Data Availability Statement

The original contributions presented in the study are included in the article/Supplementary Material; further inquiries can be directed to the corresponding author.

## Author Contributions

LH and J-YY conceived and designed the experiments. Y-TP, J-YY, Y-ZZ, BL, and PW performed the experiments. Y-TP, LL, J-YY, and BL analyzed the experiment data. LH contributed to reagents, materials, and analysis tools. Y-TP, LL, and LH wrote the paper. All authors contributed to the article and approved the submitted version.

## Funding

This research was supported by the Nature Science Foundation of China (31870631 and 32101530), Youth Programme for Natural Science Foundation of Jiangsu Province (grant no BK20181005), and Priority Academic Program Development of Jiangsu Higher Education Institutions.

## Conflict of Interest

The authors declare that the research was conducted in the absence of any commercial or financial relationships that could be construed as a potential conflict of interest.

## Publisher’s Note

All claims expressed in this article are solely those of the authors and do not necessarily represent those of their affiliated organizations, or those of the publisher, the editors and the reviewers. Any product that may be evaluated in this article, or claim that may be made by its manufacturer, is not guaranteed or endorsed by the publisher.

## Footnotes

^1^ http://genome.jgi.doe.gov/Gloci1/Gloci1.home.html

^2^ http://www.broad.mit.edu/annotation/genome/colletotrichum_gloeosporioides/home.html

## Supplementary Material

Supplementary Figure S1 Phylogenetic analysis of CgSat4 and its homologs from different fungi and identification of gene deletion mutant of CgSat4. **(A)** Phylogenetic tree of CgSat4 and its homologs constructed based on alignment of the Sat proteins from different fungi. **(B)** The predicted domains of the CgSat4. The hexagon and box indicate the S_TKc (Serine/Threonine protein kinases, catalytic) domain and a low complexity region, respectively. **(C)** The strategy of gene deletion of the *CgSAT4*. **(D)** Southern blot analysis of the WT and ∆*Cgsat4* mutant using the probes of *CgSAT4* and *HPH*, respectively. **(E)** Cellular localization of CgSat4-GFP in the conidia and vegetative hypha of *C. gloeosporioides*. **(F)** Western blot analysis of total proteins isolated from the complemented strain expressing the CgSat4-GFP fusion protein.

Supplementary Figure S2 CgSat4 does not directly interact with CgTrk1 and CgHog1. **(A)** Co-immunoprecipitation (Co-IP) assays or the interaction between CgSat4 and CgTrk1. The *CgTRK1*-GFP/*CgSAT4*-S were co-expressed in the wild-type strain. Total proteins isolated from mycelium of transformants and proteins eluted from the GFP-Trap-A beads were analyzed by Western blotting with anti-GFP and anti-S antibodies. **(B)** Yeast two hybrid assay for the interaction between CgSat4 and CgTrk1. The AD and BD plasmids were co-transformed into yeast AH109, and transformants were plated on SD-Leu-Trp for 3 d and on selective SD-Leu-Trp-His-Ade for 5 days. **(C)** Phos-tag assay analysis of CgTrk1 phosphorylation. The green fluorescence CgTrk1 (CgTrk1-GFP) fusion protein from the ∆*Cgsat4* mutant cell extracts treated with phosphatase inhibitors were subjected to Phos-tag SDS-PAGE and normal SDS-PAGE followed by immunoblotting with the anti-GFP antibody. The CgTrk1-GFP from wild-type strain cell extracts treated with phosphatase inhibitors or alkaline phosphatase CIP were used as controls. **(D)** Yeast two hybrid assay for the interaction between CgSat4 and CgHog1.
