# Supplementary material for: Involvement of Protein Kinase CgSat4 in Potassium Uptake, Cation Tolerance, and Full Virulence in Colletotrichum gloeosporioides
Source: Front Plant Sci. 2022 Apr 7;13:773898. doi: 10.3389/fpls.2022.773898 (PMC9021643; doi:10.3389/fpls.2022.773898)
Supplement: Supplementary Figure S1 — Phylogenetic analysis of CgSat4 and its homologs from different fungi and identification of gene deletion mutant of CgSat4. (A) Phylogenetic tree of CgSat4 and its homologs constructed based on alignment of the Sat proteins from different fungi. (B) The predicted domains of the CgSat4. The hexagon and box indicate the S_TKc (Serine/Threonine protein kinases, catalytic) domain and a low complexity region, respectively. (C) The strategy of gene deletion of the CgSAT4. (D) Southern blot analysis of the WT and ∆Cgsat4 mutant using the probes of CgSAT4 and HPH, respectively. (E) Cellular localization of CgSat4-GFP in the conidia and vegetative hypha of C. gloeosporioides. (F) Western blot analysis of total proteins isolated from the complemented strain expressing the CgSat4-GFP fusion protein. [file Image_1.pdf]

Supplementary FIGURE S1 Phylogenetic analysis of CgSat4 and its homologs from different fungi, identification of gene deletion mutant of *CgSAT4*, and cellular localization of CgSat4-GFP. **(A)** Phylogenetic tree of CgSat4 and its homologs constructed based on alignment of the Sat protins from different fungi. **(B)** The predicted domains of the CgSat4. The hexagon and box indicate the S_TKc (Serine/Threonine protein kinases, catalytic) domain and a low complexity region, respectively. **(C)** The strategy of gene deletion of the *CgSAT4*. **(D)** Southern blot analysis of the WT and Δ*Cgsat4* mutant using the probes of *CgSAT4* and *HPH*, respectively. **(E)** Cellular localization of CgSat4-GFP in the conidia and vegetative hypha of *C. gloeosporioides*. **(F)** Western blot analysis of total proteins isolated from the complemented strain expressing the CgSat4-GFP fusion protein.

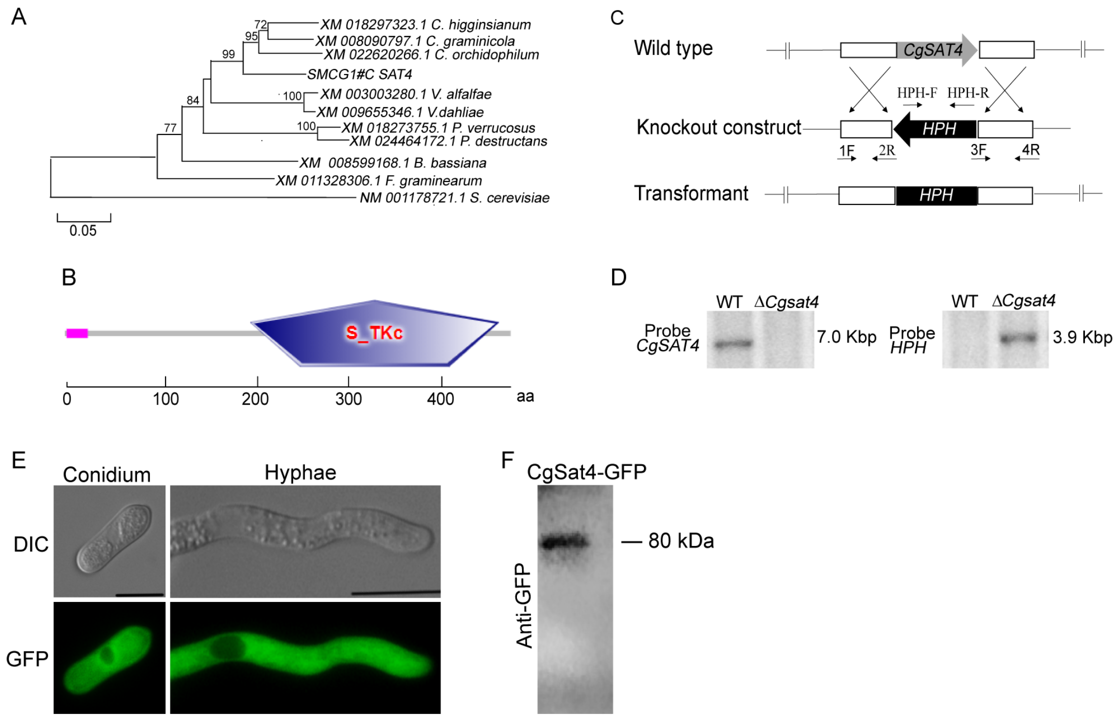

Supplementary FIGURE S2 CgSat4 is not involved in directly interacting with CgTrk1 and CgHog1, respectively, and phosphorylation of CgTrk1. **(A)** Co-immunoprecipitation (co-IP) analysis of the interaction between CgTrk1 and CgSat4. **(B)** Yeast-two-hybrid (Y2H) assay for the interaction between CgTrk1 and CgSat4. **(C)** CgTrk1 phosphorylation in the SMCG1#C and the Δ*Cgsat4* mutant. **(D)** Yeast-two-hybrid (Y2H) assay for the interaction between CgHog1 and CgSat4.

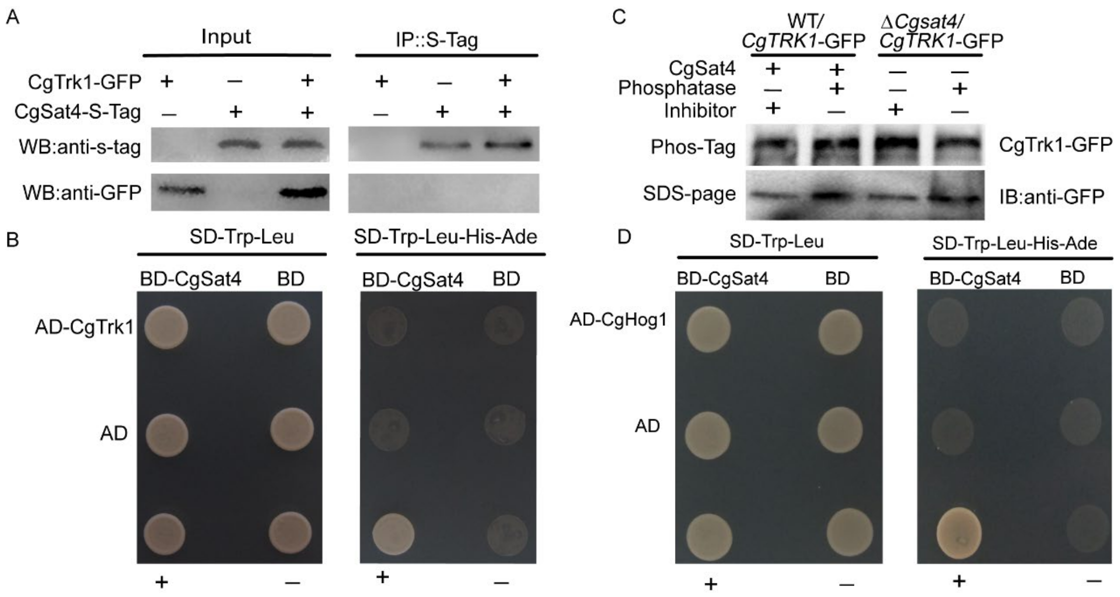
A
Input
IP::S-Tag
CgTrk1-GFP
CgSat4-S-Tag
WB:anti-s-tag
WB:anti-GFP
B
SD-Trp-Leu
SD-Trp-Leu-His-Ade
BD-CgSat4
BD
AD-CgTrk1
AD
C
WT/
CgTRK1-GFP
ΔCgsat4/
CgTRK1-GFP
CgSat4
Phosphatase
Inhibitor
Phos-Tag
SDS-page
CgTrk1-GFP
IB:anti-GFP
D
SD-Trp-Leu
SD-Trp-Leu-His-Ade
BD-CgSat4
BD
AD-CgHog1
AD
