# Supplementary material for: Involvement of Protein Kinase CgSat4 in Potassium Uptake, Cation Tolerance, and Full Virulence in Colletotrichum gloeosporioides
Source: Front Plant Sci. 2022 Apr 7;13:773898. doi: 10.3389/fpls.2022.773898 (PMC9021643; doi:10.3389/fpls.2022.773898)
Supplement: Supplementary file 2 [file Table_1.docx]

| **Supplementary Table S1. Primers used in this study** | | |
| --- | --- | --- |
| Primer | Sequence (5’ -3’) | Application |
| *CgSAT4* -1F | GAAGGGTCACCTTTACCACA | Amplify *CgSAT4* 5’ flank sequence, for gene knock out |
| *CgSAT4* - 2R | TTGACCTCCACTAGCTCCAGCCAAGCC  CAAATGCGACCGACTTGTC | Amplify *CgSAT4* 5’ flank sequence, for gene knock out  Amplify *HPH* sequence, for gene knock out |
| *HPH*-1F | GGCTTGGCTGGAGCTAGTGGAGGTCA A |  |
| *HPH*-2R | CGGTCGGCATCTACTCTATTCCTTTG | Amplify *HPH* sequence, for gene knock out  Amplify *CgSAT4* 3’ flank sequence, for gene knock out |
| *CgSAT4* -3F | CAAAGGAATAGAGTAGATGCCGAC  CGCCATCAGCCTGCTTCGATT |  |
| *CgSAT4* -4R | TGCCAAGCTTAGCAAATGC | Amplify *CgSAT4* 3’ flank sequence, for gene knock out  Amplify *CgSAT4* gene probe, for transformants screen |
| *CgSAT4* -InnerF | ATGGCGAGCCCAACAAC |  |
| *CgSAT4* -InnerR | TCAGAGACCTTCCTCACCAG | Amplify *CgSAT4* gene probe, for transformants screen  Amplify *CgSAT4* gene probe, for southern blot and transformants identification |
| *CgSAT4* -OuterF | CTTCCAGAGGCTCGTCTGC |  |
| *CgSAT4* -OuterR | GCGTCTGGCGTAGATTTCG |  |
| *CgSAT4*_SN_F | CACGAGCACCACCTCAAGTC |  |
| *CgSAT4*_SN_R | CTCATCACGACGACCCTCCAAG | Amplify *CgSAT4* gene probe, for southern blot and transformants identification  Amplification of hygromycin B phosphortransferase gene cassette, for southern blot and transformants identification |
| F1111 | GGAGGTCAACACATCAATG |  |
| F1112 | CTCTATTCCTTTGCCCTCG | Amplification of hygromycin B phosphortransferase gene cassette, for southern blot and transformants identification  For *CgSAT4*-GFP fusion construct, native promoter |
| *CgSAT4* -GFP -1F | ACTCACTATAGGGCGAATTGGGTACTCAAATTGGTTACTGCGCCGAGGCGTTCTTG |  |
| *CgSAT4*-GFP -2R | CACCACCCCGGTGAACAGCTCCTCGCCCTTGCTCACGAGACCTTCCTCACCAG | For *CgSAT4*-GFP fusion construct, native promoter  For *CgTRK1*-GFP fusion construct, native promoter |
| *CgTRK1* -GFP -1F | ACTCACTATAGGGCGAATTGGGTACTCAAATTGGTT TGGAAACGGACGCCTCC |  |
| *CgTRK1*-GFP -2R | CACCACCCCGGTGAACAGCTCCTCGCCCTTGCTCACGCGTGCTGGTGTAGTGT | For *CgTRK1*-GFP fusion construct, native promoter  Reference gene for qRT-PCR analysis |
| CgActin -1F | GTGAGGCCCTCAAAGGTAGTGG |  |
| CgActin -2R | GGATCCCAGTGCGAGACGT | Reference gene for qRT-PCR analysis  *CgTRK1* qRT-PCR analysis |
| *CgTRK1* .qPCR.1F | GCCGTCATTCTACTCTGTTACTT |  |
| *CgTRK1* .qPCR .2R | GGATTCGATGTAGTTCCCGTATT | *CgTRK1* qRT-PCR analysis  Reference gene for Yeast Two-Hybrid |
| AD-CgTrk1-F | GCCATGGAGGCCAGTGAATTC ATGCTCGACGACGCGCG |  |
| AD-CgTrk1-R | TCGAGCTCGATGGATCCC CTAGCGTGCTGGTGTAGT | Reference gene for Yeast Two-Hybrid  For CgSat4-S-Tag fusion construct, native promoter |
| BD-CgSat4-F | GCCATGGAGGCCGAATTCATGGCGAGCCCAACAACCG |  |
| BD-CgSat4-R | TGCAGGTCGACGGATCCCTCAGAGACCTTCCTCAC |  |
| AD-CgHog1-F | GCCATGGAGGCCAGTGAATTC ATGGCGGAATTCATCAGAG |  |
| AD-CgHog1-R | TCGAGCTCGATGGATCCCCTATTGCCCGTTGAATT |  |
| CgSat4-S-Tag-F | ACTCACTATAGGGCGAATTGGGTACTCAAATTGGTTCATATCGAATCTCTGGCGGT |  |
| CgSat4-S-Tag-R | TTCGAATTTAGCAGCAGCGGTTTCTTTATGAGACCTTCCTCACCAGCCT | For CgSat4-S-Tag fusion construct, native promoter |
